# Gait Trajectory Prediction on an Embedded Microcontroller Using Deep Learning

**DOI:** 10.3390/s22218441

**Published:** 2022-11-03

**Authors:** Mohamed Karakish, Moustafa A. Fouz, Ahmed ELsawaf

**Affiliations:** 1 Mechanical Engineering Department, College of Engineering and Technology, Cairo Campus, Arab Academy for Science, Technology and Maritime Transport (AASTMT), Cairo 11757, Egypt; 2 Faculty of Engineering, German International University, Cairo, Egypt

**Keywords:** gait trajectory prediction, deep learning, MLP, CNN, embedded system, microcontroller, TensorFlow Lite micro, ESP32

## Abstract

Achieving a normal gait trajectory for an amputee’s active prosthesis is challenging due to its kinematic complexity. Accordingly, lower limb gait trajectory kinematics and gait phase segmentation are essential parameters in controlling an active prosthesis. Recently, the most practiced algorithm in gait trajectory generation is the neural network. Deploying such a complex Artificial Neural Network (ANN) algorithm on an embedded system requires performing the calculations on an external computational device; however, this approach lacks mobility and reliability. In this paper, more simple and reliable ANNs are investigated to be deployed on a single low-cost Microcontroller (MC) and hence provide system mobility. Two neural network configurations were studied: Multi-Layered Perceptron (MLP) and Convolutional Neural Network (CNN); the models were trained on shank and foot IMU data. The data were collected from four subjects and tested on a fifth to predict the trajectory of 200 ms ahead. The prediction was made for two cases: with and without providing the current phase of the gait. Then, the models were deployed on a low-cost microcontroller (ESP32). It was found that with fewer data (excluding the current gait phase), CNN achieved a better correlation coefficient of 0.973 when compared to 0.945 for MLP; when including the current phase, both network configurations achieved better correlation coefficients of nearly 0.98. However, when comparing the execution time required for the prediction on the intended MC, MLP was much faster than CNN, with an execution time of 2.4 ms and 142 ms, respectively. In summary, it was found that when training data are scarce, CNN is more efficient within the acceptable execution time, while MLP achieves relative accuracy with low execution time with enough data.

## 1. Introduction

Walking is an essential activity for human locomotion. However, it is a very complex process, and any disorder, instability, or incoordination can lead to deviations and challenges during locomotion [1]. Furthermore, gait deviations faced by lower-limb amputee patients caused by prosthetics are critical in the long term. These deviations are caused by the asymmetry or the non-repeatability differences between a healthy person and an amputee’s gait [2]. As a result, these anomalies can increase the energy cost during motion, overload the muscles, and cause damage to joint structures and skin. Moreover, to avert these complications, lower limb kinematics are a critical factor when designing active intelligent prostheses [3,4].

Various techniques for different applications are adopted to analyze gait kinematics and kinetics [5]. The first technique is motion capture systems, utilizing infrared cameras with reflective markers [6,7,8]. In addition, relatively low-cost non-marker-based image processing using time of flight (TOF) cameras in motion tracking, such as in Microsoft Kinect, are used in this method [7,9]. The second technique uses inertial measurement units (IMU) by positioning the IMU sensor on different body parts and measuring the kinematic parameters (angle, angular velocity and acceleration) for each joint (hip, knee, and ankle) and generates a 3D kinematic model used for gait analysis [2,7,10,11,12,13]. The third type uses an array of force sensors under the foot to measure the ground reaction force (GRF) and its the distribution on foot. Using the scalar of the reaction force and the center of the reaction force gait cycle can be recreated [7,14]. Finally, electromyography is used to measure the contraction of the main lower limb muscles—hip muscles, shank, and ankle muscles [15]—using surface-mounted electrodes to plot the kinematic model of the gait cycle [7].

From 2010 to 2020, the sensory-based publications on lower limb kinematics signal recording focused on using wearable sensors by nearly 79% of the publications, such as IMU sensors, force sensors, and EMG sensors, while only 12% used vision-based and other techniques [1,16]. Vu et al. [1] conducted a study on detecting gait phase and found that the IMU was the most commonly used sensor in gait analysis phase detection by nearly 78% of the publications compared to 14% for force and 8% for EMG sensors. Although force sensors are relatively low cost and show high precision in gait analysis [17], the sensor’s signal needs to be filtered to remove the noise that can affect the results. Furthermore, due to the dynamic load exerted on the sensor by the foot during the gait cycle, the expected lifespan of the sensor can be short due to the mechanical wear [1]. On the other hand, EMG sensors have become less prominent due to their complexity of usage in data acquisition, processing [18], and for its sensitivity to any substance trapped between the probe and the skin, such as moisture [1]. Furthermore, the IMU sensor module is relatively cheap, reliable, has low power consumption, and can be easily positioned on the body. It consists of three sensors: an accelerometer, a gyroscope, and a magnetometer used to measure angular velocity and linear acceleration for the gait cycle analysis.

There is a progressive focus on analyzing the human gait using different techniques; each has its benefits and drawbacks according to the application [7]. Extracting the gait motion characteristics helps in detecting gait deviations which can be an indication of a possibility of tripping, slipping, or balance loss [19,20,21,22,23]. Alternatively, it can compensate for the delay of the response time of the control system [24,25,26]. Moreover, the lower limb’s future trajectory prediction can be used to solve numerous problems facing robotic lower limb prothesis/orthosis. Furthermore, detecting the current phase in the gait cycle can benefit the assistive powered prostheses control. The gait phase has the required information to be able to determine the needed angle, angular velocity, and torque, which can improve the performance of the controller by providing the current gait phase [27,28,29]. As a result, better control has an effect on the patient, which can help with reducing the energy cost of walking with a powered limb [30]. The human gait cycle can be segmented into two main phases (stance phase and swing phase), four phases (initial contact, foot flat, heel off, and toe-off) or even seven phases (loading response, mid stance, terminal stance, pre swing, initial swing, mid swing, and terminal swing) [17,31].

From the previous research, it was found that the LSTM network achieved the best results in time-series data prediction [32] and especially in detecting human activity recognition or predicting human gait cycle kinematics. However, we try to achieve an embedded system in a prosthesis that does not depend on an external computing source by any means, wired or wireless (PC, servers, …, etc.), that is as reliable as possible, by implementing an LSTM network on an embedded system microcontroller to detect human activity.

This study aims to develop a deep MLP and CNN model to predict a future frame of gait trajectory. Furthermore, we investigate the capability of MLP and CNN in handling sequential data with an accuracy comparable to that of a long short-term memory (LSTM) neural network. This approach targets using the current and previous sensor readings to predict the future gait trajectory window while using new readings for each prediction to avoid the accumulation of error. Moreover, we study and compare the accuracy of regression (forecasting) of both neural network configurations while maintaining an acceptable computational time. What is more, we study the capability of a low-cost, low-power microcontroller in implementing both models while achieving a good inference time on the targeted hardware.

This article is organized as follows. Firstly, Section 2 contains the previous related research for each part of this study. Next, Section 3 contains the materials and methods, discussing the used dataset and its properties, data processing, developed machine learning algorithms and prediction performance evaluation methods. Then, Section 4 shows the results from the trained models and a comparison between the used methods. Then, Section 5 has a discussion of the results. Finally, Section 6 includes the conclusion of this study.

## 2. Related Work

In this study, the first part is how to capture the motion parameters of a person. Ahmedi et al. [33] indicated the possibility of the reconstruction of a 3D gait kinematic model with efficient computation using seven IMU and foot force sensors. Furthermore, Hu et al. [34] proposed a method to estimate the joint angles of lower limbs (i.e., hip, knee and ankle angles) using the minimal number of IMUs of only four sensors. Mishra et al. [35] and Yin et al. [36] used the surface EMG signals of EMG sensors on different muscles in gait analysis and measured speed to develop EMG-driven speed-control for exoskeleton motion control.

The second area is gait kinematic trajectory future windows prediction. Binbin Su et al. [37] used an LSMT neural network to predict lower body segment trajectory (angular velocity of the thigh, shank and foot) up to 200 ms (10 time frame) ahead in the future based on past observations up to 600 ms (30 time frame), and they achieved a z-score normalized angular velocity error of 0.005, MAE of 0.299, RMSE of 0.487 and Coefficient of Determination of 0.91 for the inter-subject’s foot trajectory. Furthermore, Zaroug et al. [38] used different LSTM neural network architectures (Vanilla, Stacked, Bidirectional and Autoencoders) in predicting lower limb kinematic parameters (angular velocity and linear acceleration of the thigh, shank and foot) up to 100 ms (five time frames) ahead, and they reported that the best result was achieved using autoencoder LSTM architecture with a normalized angular velocity MAE of 0.276 and RMSE of 0.419 for the inter-subject’s foot trajectory. Zaroug et al. also [39] used an autoencoder LSTM neural network architecture to predict lower body segment trajectory (angular velocity of the thigh, shank) up to five samples frontwards using a previous window size of 25 samples, and they attained a normalized angular velocity MAE of 0.28, MSE of 0.001 and Correlation Coefficient between the predicted and actual angular velocity of 0.99 for the inter-subject’s thigh trajectory, and they attained an MAE of 0.24, MSE of 0.001 and Correlation Coefficient of 0.99 for the inter-subject’s shank trajectory. Additionally, Sun et al. [12] proved that a feed-forward neural network is helpful in time sequence data and capable of predicting IMU human gait kinematic parameters (acceleration and angular velocity) based on readings from the IMU from other body parts, obtaining a Correlation Coefficient of 0.89 for predicting the angular velocity of the shank using only the IMU readings from the foot and a Correlation Coefficient of 0.8 for predicting the angular velocity of the thigh using only the IMU readings from the foot while using a window of size five-time frames.

The third area involves studying the effect of the current gait phase on the accuracy of the trained models. In most research for gait analysis, a four-phase model is commonly used so that the gait is partitioned into: (a) the initial foot contact (IC) with the ground or Heel Strike (HS); (b) the loading response phase or Flat Foot (FF); (c) the heel lifting or Heel-Off (HO); and (d) the initial Swing Phase (SP) or Toe-Off (TO). However, Taborri et al. [40] proved that a two-phase model had the sufficient data to control the knee module of an active orthosis. For that, the gait was segmented into two phases, (a) Swing Phase (SW); and (b) Stance Phase (ST), to avoid any computational complexity due to hardware limitations. Cho et al. [41] compared two-phase segmentation methods, first using a camera-based system and the other using an IMU-based system, while observing the sagittal, frontal, and transverse planes for body joints. This method proved that the IMU system could be reliable and be used in gait phase segmentation. In the same study mentioned before, Binbin Su et al. [37] used an LSTM neural network architecture to detect five phases of the gait cycle (loading response, mid-stance, terminal stance, pre-swing, and swing) up to 200 ms (10 time frames) ahead in the future based on past observations up to 600 ms (30 time frames), and they achieved a detection accuracy of 79% for the loading response phase, 87% for the mid-stance phase, 77% for the terminal stance phase, 85% for the pre-swing phase, and 95% for the swing phase for the inter-subject test. To demonstrate the potential of CNNs, it have been used for tasks such as autonomously detecting human activities using the accelerometer sensor raw data from fitness equipment and smartphones. Yang et al. [42] used the Deep Convolutional Neural Network to classify human activities based on time-series readings and stated that the benefits of using DCNN in this application are: feature extraction can be performed by CNN automatically by using raw input data, and feature extraction and classification can be performed on a single CNN model, which can be less hardware intensive than dividing both tasks on more than one model. Furthermore, Lee et al. [14] used a smart insole with on-board sensors (pressure sensor, accelerometer, and gyroscope sensor) and DCNN neural network architecture to detect seven gait phases for seven gait types (walking, fast walking, running, stair climbing, stair descending, hill climbing, and hill descending) and proved that the best classification rate could be achieved by using the three sensors and could achieve a 94% total classification rate using the data of five window step input sensors data.

Finally, the main aim of the study to validate the capability of a microcontroller in the predection of future gait trajectory in viable time. Alessandrini et al. [10] achieved an accuracy and reached 95.54% by applying a trained biLSTM network on an STM32L476RG microcontroller, when observing the load of the neural network on the hardware of the microcontroller. Furthermore, it was found that the network could need more than 100% of the available RAM, and the inference time could reach 150 ms to achieve the best accuracy.

## 3. Materials and Methods

### 3.1. Dataset and Its Properties

**Figure 1:**
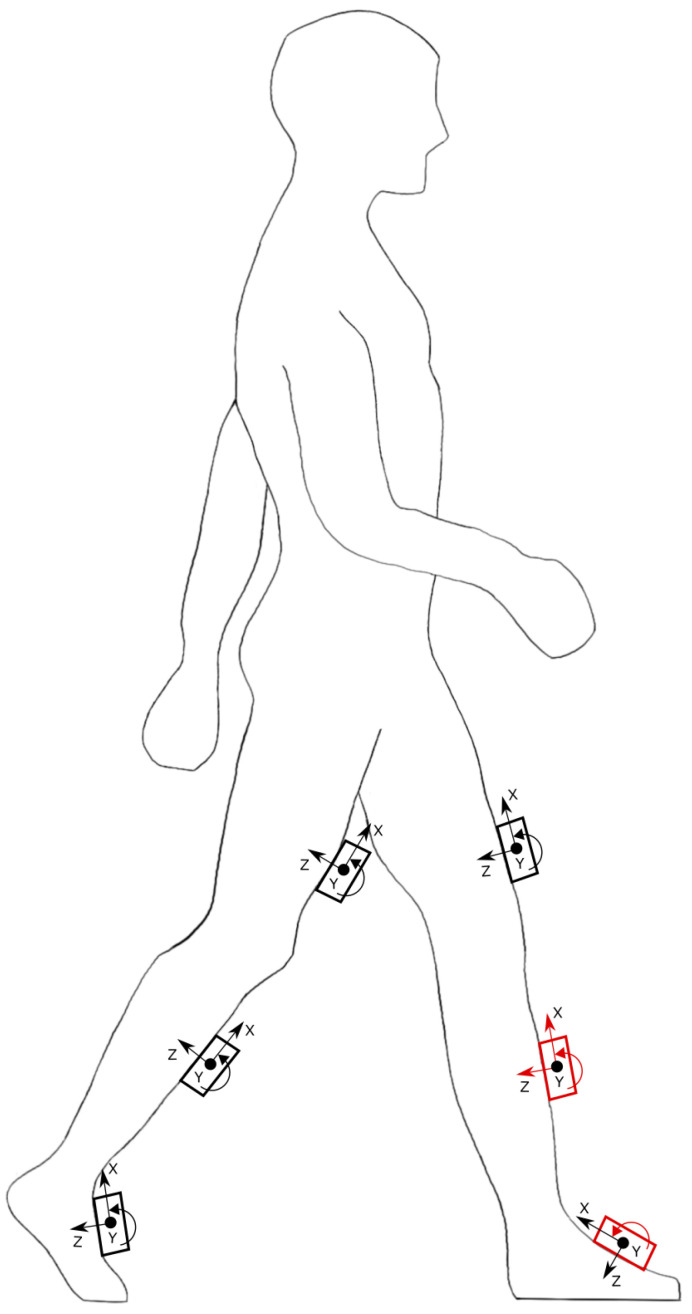
Location of inertial sensors position on the lower limbs (black) and the used sensors’ readings (red).

In this research, the human gait database (HuGaDB) for activity recognition from wearable inertial sensors was used, which was published by Chereshnev et al. [43] in order to help the community research gait analysis for both human activity recognition and how are they performed. At the same time, it is also used to understand the movement of the different parts of the lower limb, each on its own and relative to the other parts, while performing different activities such as walking, running, and going up and down stairs. Furthermore, the dataset consists of readings from six different MPU9250 inertial sensors, two of them placed on the rectus femoris muscle five centimeters above the knee, the other two installed around the middle of the shinbone at the level where the calf ends, and the last pair positioned on the feet on the metatarsal bones, as illustrated in Figure 1.

The IMU’s accelerometer and gyroscope data collection was completed on 18 healthy young adults: 4 females and 14 males, with an average age of 23.67 (std: 3.69) years, an average height of 179.06 (std: 9.85) cm and an average weight of 73.44 (std: 16.67) kg. The contributors performed different activities—sitting, standing up, walking, going up the stairs, walking, sitting down—at different speeds, without any obstacles in their way. Each activity was repeated for each participant, and during the activity, the data from the sensors were recorded, giving a total of 2,111,962 samples.

### 3.2. Data Prepossessing

Walking data were used from the dataset, which was recorded while walking at various speeds and turning at the end of the test course. These variations in the recorded gait can result in a more generically trained neural network that is not focused on a certain walking speed. In addition, the problem with the dataset is that some gyroscope readings were corrupted due to the amplification of the data, which led to clipping of the sensor signal. Only the data from the four subjects whose gyroscope readings had not been corrupted were used in the research.

The data of four kinematic parameters from the left leg were used on the unilateral amputee’s active prophesies: accelerometer values of the shank along the x and z-axes, and gyroscope values of both the shank and the foot about the y-axis, as shown in Table 1 and Figure 1.

**Figure 2:**
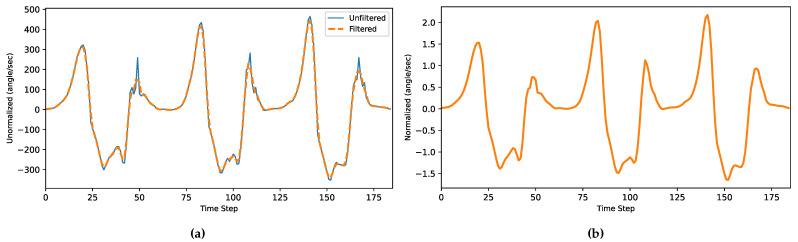
(**a**) Filtered data. (**b**) Normalized filtered data (moving average window = 3).

The dataset was made from the raw readings recorded from the sensors. A windowed moving average filter (MAF) was used to filter the data of both the gyroscope and accelerometer [44]. This filter was used in order to counter the bias drift of the inertial sensors [45], which can be represented by the following equation [46]:

(1) $$z\left(n\right)=\frac{1}{P+1}\sum_{j=0}^{P}x\left(n-j\right)$$

where z(n) is the output filtered data, while the input unfiltered data is *x*, and *P* is the length of the window. The more samples P+1 averaged over, the smoother and more delayed the output. The delay occurs because the output z(n) is a function of only the current and previous inputs x(n−j), 0≤j≤P, filtered data with an averaging window of 3, as shown in Figure 2a. The moving average value was chosen to avoid over smoothing and losing important features in the signal. Then, the samples were normalized to mean zero and unit variance using z-score normalization, as shown in Figure 2b.

### 3.3. Time-Series Sequential Data into Supervised Learning Frames Transformation

**Figure 3:**
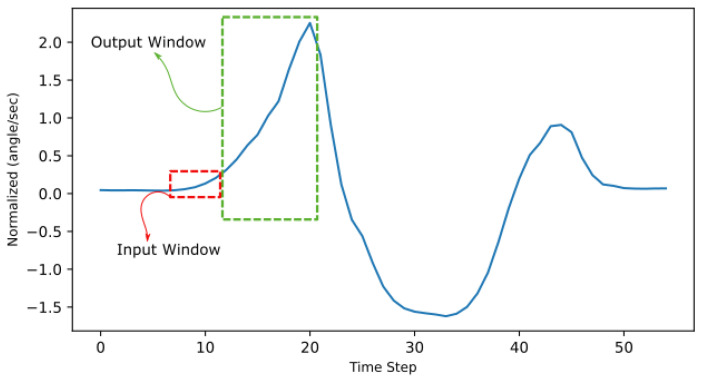
Sliding window illustration on the ankle’s normalized angular velocity data (1 feature). The input window of n samples and output windows of z samples, where n=5 and z=10.

For the human gait cycle kinematic shown in Table 1, parameters are time-series data which are either linear acceleration or angular velocity sampled over fixed time intervals. Moreover, the four parallel readings or four variable features were received from the two IMUs. However, before supplying this recorded time series of IMU data to a neural network algorithm, the shape of the parallel data should be transformed into a suitable matrix for a supervised machine learning algorithm. For M×N, where *M* is the number of samples, and *N* is the number of features transformed into a 3D dataset using a sliding window, each layer has a 2D dimension of an M×N data sample, and the depth of the dataset depends on the number of samples to which the sliding window will be applied [47]. A sliding window is used to phrase the time-series data into a supervised learning format by using the previous time step’s value (z−n) to forecast the subsequent time step’s value (z−m) [48], as illustrated in Figure 3.

### 3.4. Machine Learning Models for Phase Segmentation and Trajectory Generation

In time-series data, forecasting or predicting the output can be a complex task due to the temporal dependence between the data. Moreover, complicated time-series forecasting issues with multiple input variables, complex nonlinear relationships, and missing data can be solved using machine learning methods [47]. In addition, the neural network is required to process thousands of readings for multiple features. Thus, to predict such complex data with the limited computational load of the MC, an adequate DNN is investigated.

#### 3.4.1. Deep Multi-Layer Perceptron

**Figure 4:**
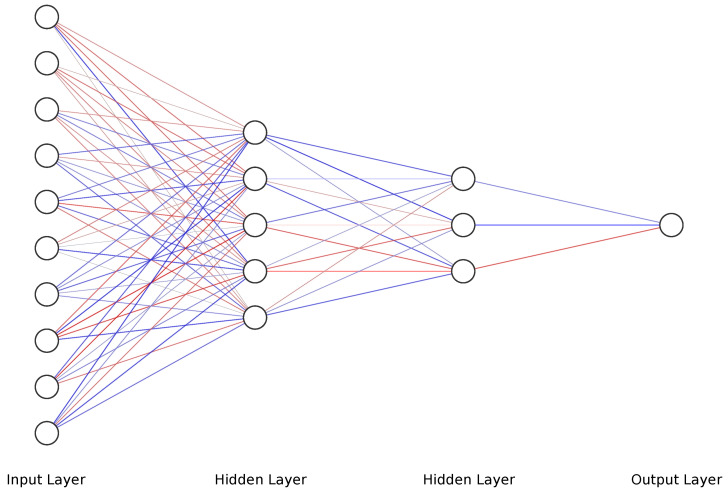
DMLP Neural Network Diagram.

Multi-Layer Perceptrons, or MLPs, are less complex with a computational load. Moreover, MLPs approximate a mapping function between input and output, including an input layer, an output layer, and at least one hidden layer. In the hidden layer, the neurons are connected to the neurons in the previous and the following layers, as shown in Figure 4. In the training process, the weights and bias parameters in the hidden layers are adjusted continuously to make the output value from the output layer consistent with the actual value from the dataset. MLPs are valuable for time-series analysis for many reasons, such as being robust to noise, non-linearity, applicable for multi-input multi-output (MIMOS) configuration, and able to predict multi-step output [49]. Rectified linear units (ReLU) were used in the hidden nodes, the input nodes, and output nodes, which has a constant gradient when x>0, while it is null for x<0, as shown in Equation (3).

There are two weight matrices, *W* and *H*, and two corresponding bias vectors, *b* and *c*. If there are m hidden neurons, ${\bar{x}}_{i}\in R^{n\times 1}$ (input layer), and ${\bar{y}}_{i}\in R^{k\times 1}$ (output layer), the dynamics are defined by the following transformations [50]:

(2) $$\left\{\begin{array}{cc} \bar{z}=f_{h}\left(W^{T}\bar{x}+\bar{b}\right) & \;\mathrm{where}\;W\in R^{n\times m}\;\mathrm{and}\;\bar{b}\in R^{m\times 1}\;\\ \bar{y}=f_{a}\left(H^{T}\bar{z}+\bar{c}\right) & \;\mathrm{where}\;H\in R^{m\times k}\;\mathrm{and}\;\bar{c}\in R^{k\times 1} \end{array}\right.$$

(3) $$f_{ReLU}\left(x\right)=\max\left(0,x\right)$$

Deep MLP was used mainly as it has a lower computational load compared to other NN models when deploying the trained model [51], which is crucial due to the limited resources of the microcontroller. The configuration of the architecture of DMLP was optimized by the Grid Search Algorithm to find the best number of layers and neurons in each layer while considering both the size and the accuracy of the model. It may be a heavy computational algorithm, but it will ensure the best possible configuration, and in this case, the tuning was completed on PC before deploying on a microcontroller. For that, the computational load/time is not of concern at this point in comparison with the model accuracy [52]. The concluded configuration can be found in Table 2.

#### 3.4.2. Deep Convolutional Neural Network

In neural network applications for image processing, a technique known as convolutional neural networks (CNNs) is used. In challenging computer vision issues, they have demonstrated their effectiveness by reaching state-of-the-art results on tasks such as picture classification and by serving as a component in hybrid models for entirely new problems such as object localization and image captioning [48]. Using CNNs for time-series forecasting makes use of their ability to learn and automatically extract characteristics from large amounts of unstructured input data [53]. A sequence of observations can be considered as a one-dimensional image that a CNN model can interpret and distil into the most critical features’ aspects.

**Figure 5:**
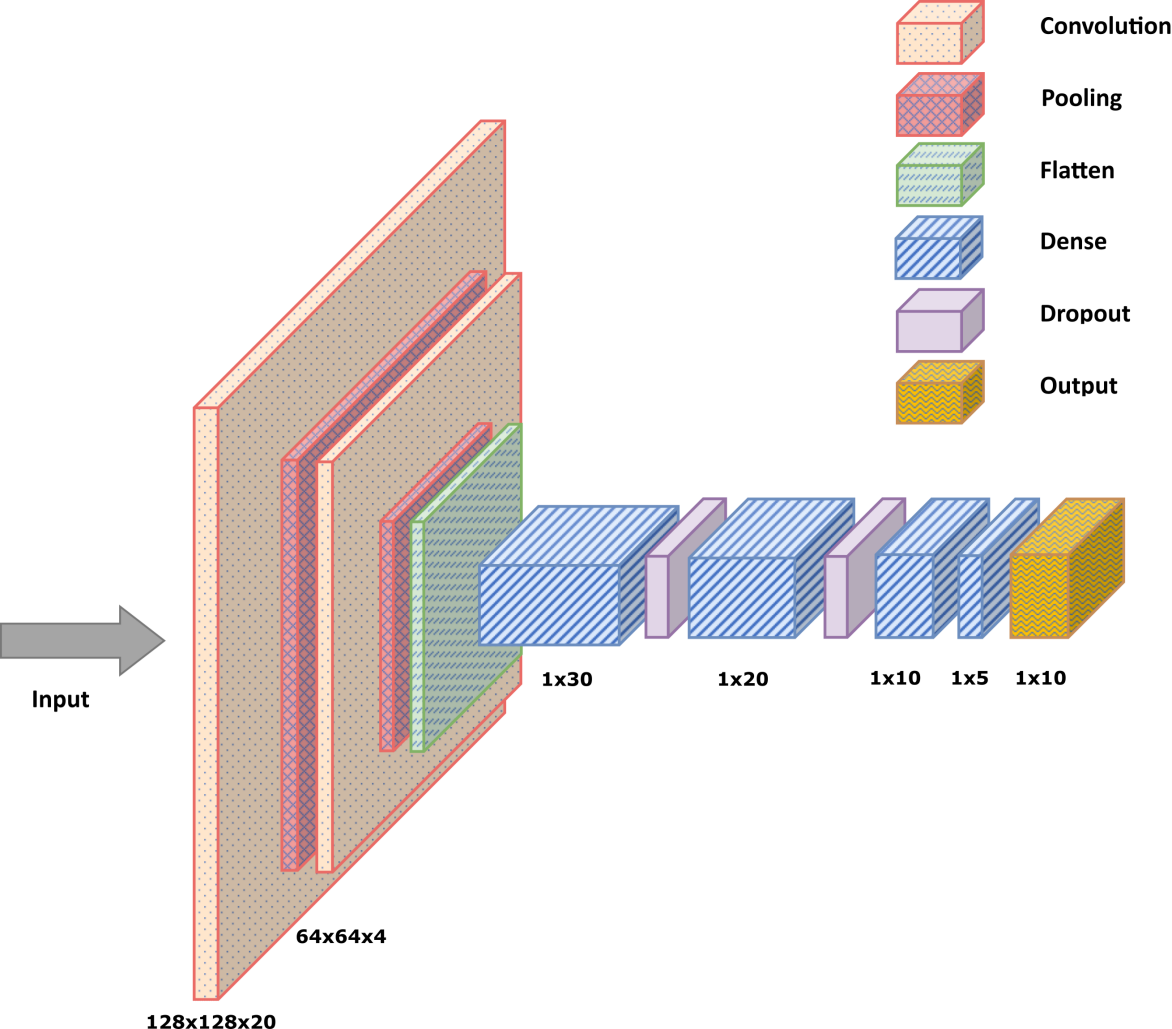
DCNN Neural Network.

Primarily, CNN architecture is inspired by visual neuro-science and has convolutional layers and pooling layers at its initial stages. Fully connected layers were implemented in sequence with the convolutional and pooling layers. After that, the extracted features are provided into dense layers—either fully connected or not—to output a vector whose dimension is the same as the number of classes, as illustrated in Figure 5.

A convolutional layer shows the idea of local receptive fields and shared weights. It often includes several feature maps, and each map is filtered through a shared convolutional kernel; then, the locally weighted sum is activated through a nonlinear active function. For a 1D convolutional layer, suppose the input of the convolutional layer is $v\in R^{A\times B}$, where B is the number of bands, and A is the length of the inputs in each band. The output of the convolutional layer is [54]:

(4) $$h_{j,k}=f\left(\sum_{b=1}^{s}w_{b,j}^{T}v_{k+b-1}+a_{j}\right)$$

where $h_{j,k}$ is the value on the kth output band on the jth feature map, s is the filter size, $w_{b,j}$ is the weight vector of the bth band of the jth filter, $a_{j}$ is the bias of the jth feature map, and f is the active function.

The developed CNN model contains 15 layers. The model has four convolution layers, and every two layers is followed by a max-pooling layer. The convolution layers use Rectified Linear Unit (ReLU) as the active function, as shown in Equation (3). The last four layers are not fully connected layers with a droupout layer between each dense with a dropout rate of 0.01. The activation function of the second not fully connected layer is also a ReLU function. The configuration of the architecture of DCNN was optimized by the Grid Search Algorithm to find the best number of layers and neurons in each layer while considering the load on the microcontroller; the used configuration can be found in detail in Table 3.

### 3.5. Neural Network Model Gait Kinematics Prediction Performance Evaluation

Gait cycle kinematic parameters are numeric values, which can be considered a regression problem. There are a lot of statistical tools to evaluate the performance of the regression model by comparing the predicted gait trajectory with the actual trajectory. The most common methods based on the previous research to evaluate the generated trajectory are as follows [55]:

The evaluation methodology considers the coincidence of the predicted trajectory ${\hat{y}}_{j}$ with respect to the actual trajectory $y_{j}$ over *n* time-steps.

1. Bias
  (5) $$Bias=\frac{1}{n}\sum_{j=1}^{n}\left(y_{j}-{\hat{y}}_{j}\right)$$
  Bias shows the tendency of the model to either over-predict or under-predict the trajectory relative to the actual one.
2. Mean Absolute Error
  (6) $$MAE=\frac{1}{n}\sum_{j=1}^{n}\left|y_{j}-{\hat{y}}_{j}\right|$$
  Mean Absolute Error is the average of the absolute prediction error values, which shows the average error in prediction regardless of its polarity.
3. Root Mean Squared Error
  (7) $$RMSE=\sqrt{\frac{1}{n}\sum_{j=1}^{n}{\left(y_{j}-{\hat{y}}_{j}\right)}^{2}}$$
  Root Mean Squared Error is the root of the average of the squared prediction error, which shows the average error in prediction regardless of its polarity using squaring instead of absolute.
4. Coefficient of Determination (R Squared—$R^{2}$)
  (8) $$R^{2}=1-\frac{\sum_{j=1}^{n}{\left(P_{j}-A_{j}\right)}^{2}}{\sum_{j=1}^{n}{\left(A_{j}-\bar{A}\right)}^{2}}$$
  Coefficient of Determination indicates the performance of the model or the goodness of the fitting. As the $R^{2}$ value tends to 1, it indicates a better prediction, which can help with comparing the performance of models.
5. Pearson correlation coefficient
  (9) $$CC=\frac{\mathrm{cov}(y,\hat{y})}{\sigma_{y}\sigma_{\hat{y}}}$$
  where cov(y) is the covariance between the predicted and actual trajectory value. Meanwhile, $\sigma_{y}$, $\sigma_{\hat{y}}$ are the standard deviations for *y*.
  Pearson correlation coefficient indicates the accuracy of the model. As the predicted and actual value tends to form a linear correlation, it indicates a better prediction. For that, as the correlation coefficient value tends to 1, that means that the model predicts better values that are closer to the actual ones.

### 3.6. Phase Segmentation

A different approach was implemented; an extra feature—gait phase segmentation— with the kinematic data was provided during the training stage to improve the underdevelopment model’s accuracy and computation load.

**Figure 6:**
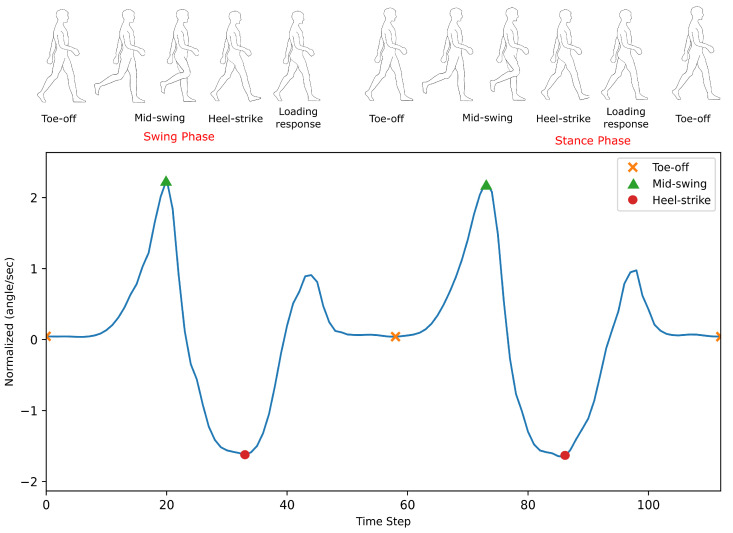
Gait Phase Segments (1 TS = 20 ms).

In testing, we can see the effect of presenting the model with more information on both the accuracy of trajectory prediction and computational load. The current phase state was detected and provided to the model in the second test, and statistical analysis was completed on the predicted trajectory and compared to the first test without the phase information. The gait was segmented using the threshold method using the ankle’s IMU angular velocity reading to determine the current phase: either the swing phase or the stance phase (Figure 6).

## 4. Results

### 4.1. Trained MLP and CNN Testing Results

**Figure 7:**
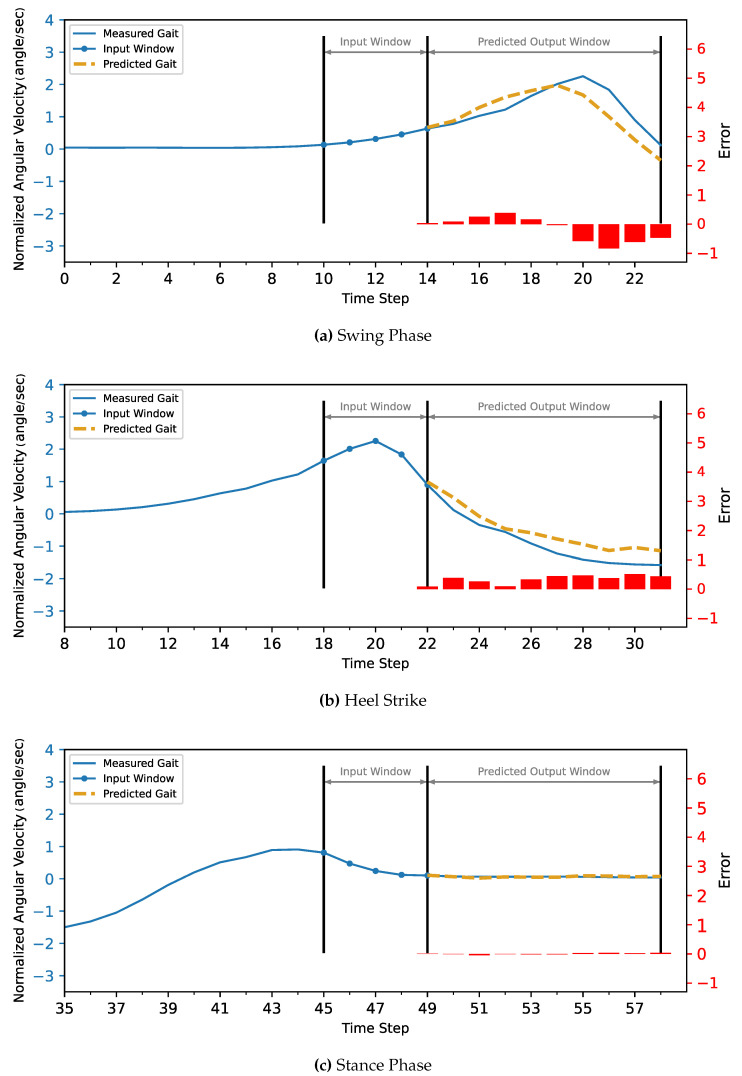
The MLP model without CGP provided 10 time steps ahead prediction for z-score normalized angular velocity of the foot of the inter-subject. The angular velocities are normalized to have zero mean and unit variance. The actual gait is shown in blue, and the predicted trajectory is shown in gold (dashed).

**Figure 8:**
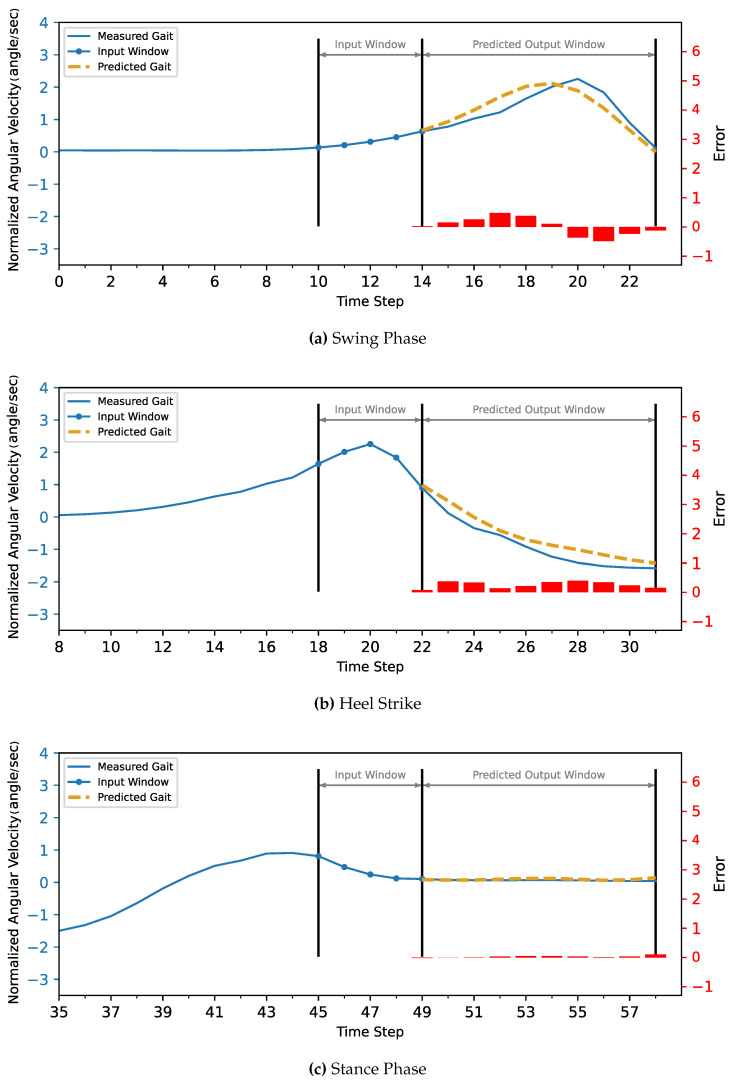
The CNN model without CGP provided 10 time steps ahead prediction for z-score normalized angular velocity of the foot of the inter-subject. The angular velocities are normalized to have zero mean and unit variance. The actual gait is shown in blue, and the predicted trajectory is shown in gold (dashed).

**Figure 9:**
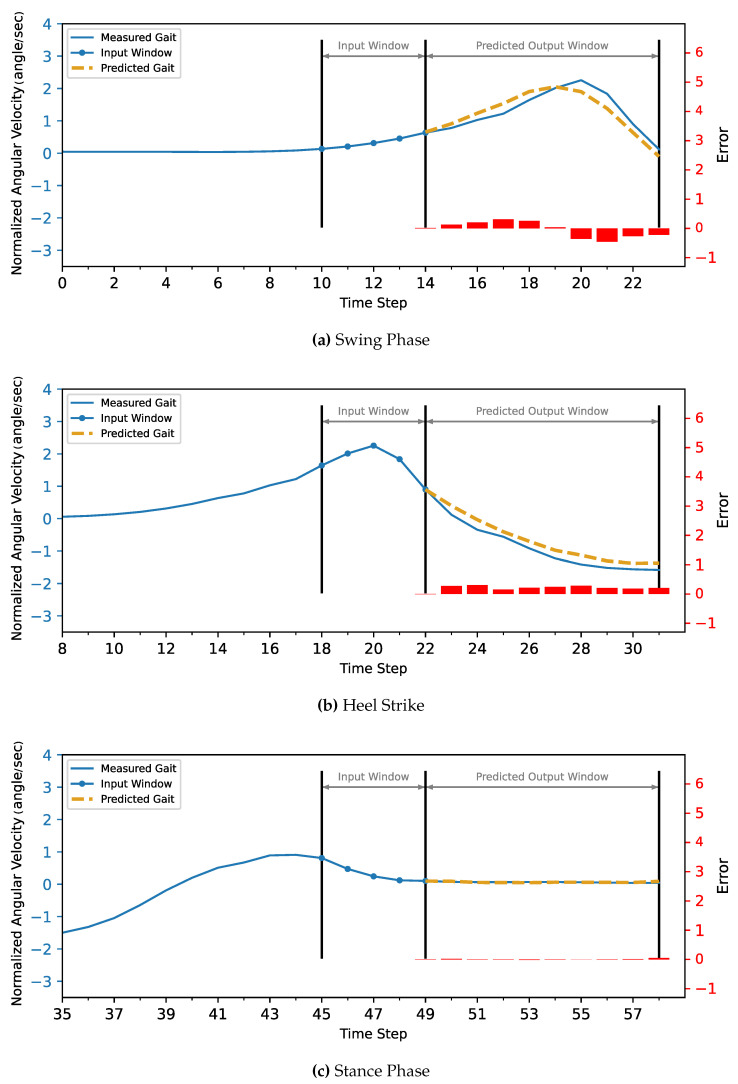
The MLP model with CGP provided 10 time steps ahead prediction for z-score normalized angular velocity of the foot of the inter-subject. The angular velocities are normalized to have zero mean and unit variance. The actual gait is shown in blue, and the predicted trajectory is shown in gold (dashed).

**Figure 10:**
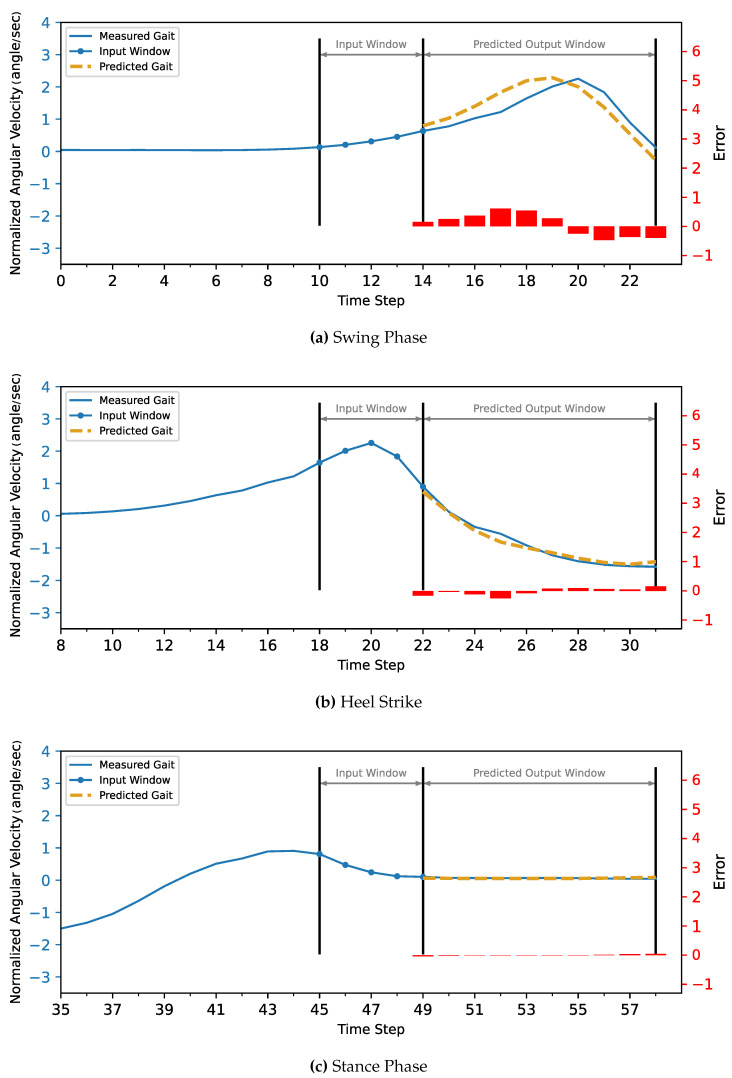
The CNN model with CGP 10 time steps ahead prediction for z-score normalized angular velocity of the foot of the inter-subject. The angular velocities are normalized to have zero mean and unit variance. The actual gait is shown in blue, and the predicted trajectory is shown in gold (dashed).

**Figure 11:**
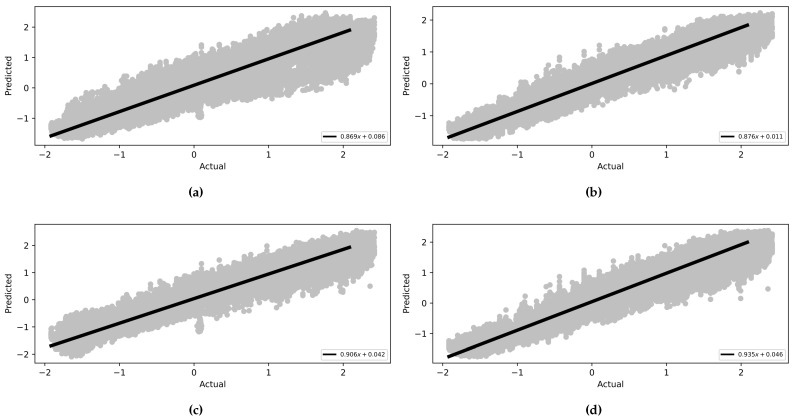
Linear correlation between the actual values (x-axis) and the predicted values (y-axis) for foot angular velocity of the 5th subject. (**a**) MLP—no CGP. (**b**) MLP—CGP. (**c**) CNN—no CGP. (**d**) CNN—CGP.

The MLP and CNN architecture models were coded in Python 3, using the TensorFlow library. The models were trained and tested on a laptop computer with a CPU Ryzen 9 5950X, GPU RTX 3070, and 32 GB of RAM before being deployed to the ESP32 microcontroller. The models’ hyperparameters were determined first with a Grid Search Algorithm, including the number of layers, the number of neurons in each layer, batch size, and the number of epochs. Then, the optimum configuration was manually optimized by sacrificing the most achieved accuracy to minimize both the inference time and model size to be suitable for the microcontroller’s hardware (CPU and memory). Due to the stochastic nature of the algorithms, the models were retrained for the same configuration until they achieved the best results.The optimized model was trained for 1000 epochs with an early stop if the performance did not increase for 20 consecutive epochs. The model fitting evaluated the performance based on the Mean Squared Error for the gait trajectory regression. The models were trained twice, the first training without providing the info about the CGP shown in Figure 7 and Figure 8, and the second training, the current phase (Swing Phase or Stance Phase), was provided in the training data in Figure 9 and Figure 10. The models are required in both training cases to estimate angular velocity ten time frames ahead based on the previous five time frames. The model was trained on the kinematic readings of the shanks of four subjects and tested on predicting a 5th one to test the generalization of the prediction for unseen subjects, and the statistical results of the first and second training methods are shown in Table 4 and Figure 11.

### 4.2. Microcontroller Inference Test

After training the models on PC, using tinymlgen library based on TensorFlow Micro Lite, the models were exported using tensorflow standard tools into a C source file that contains the TensorFlow Lite model as a char array, which was deployed on the microcontroller. The MCU used is ESP32, which has 32 bit dual core processor speeds up to 240 MHz, 512 Kb ram, and 4 MB flash memory. The computation cost of the used algorithm has been measured by feeding the 5th subject kinematic data into the MCU. Afterwards, we calculate the execution time and memory utilization to the complete the prediction; the results can be found in Table 5.

## 5. Discussion

In this study, both neural network configurations were tested for predicting the angular velocity of the foot using angular velocity and linear acceleration from IMUs mounted on the shank and an optional input CGP whether the current reading is during the swing or the stance phase in the gait cycle. Previous studies used neural networks to perform predictions for the preceding time frame. However, in this study, the algorithms predict the trajectory of a 10 time frame, or nearly 200 ms ahead, which represents almost 20% of a full stride. The data were used for daily walking with different speeds, collected from five subjects, four of which were used for training and the fifth was used for testing.

The models are compared with other publications’ findings based on the best accuracy while maintaining the size of the network due to the limitations of the hardware capabilities. The trained models could achieve good results even after reducing the size of the networks. The trained MLP and CNN achieved a root mean squared error of 0.298 and 0.245 (deg/s) and with CGP 0.226 and 0.217 (deg/s) for foot trajectory 200 ms ahead, while Su Binbin et al. [37] achieved a difference in RMSE of +0.27 (deg/s) for inter-subject implementation 200 ms ahead using an LSTM network. Zroug et al. [38] achieved a difference of +0.202 ± 0.25 (deg/s) using the ED-LSTM configuration. Another parameter has been considered is the linearity between the actual and predicted values. The trained MLP and CNN has achieved a correlation coefficient of 0.945 and 0.973 (deg/s) and with phase 0.979 and 0.979 (deg/s), while Su Binbin et al. achieved a CC with difference of −0.069 for an inter-subject test and Zroug et al. achieved a CC of −0.089 ± 0.14; see Table 6 for the other publications’ results.

The framework of this research project is to develop an embedded active prothesis system. Therefore, it is more logically to use a system of built IMUs for both training and deploying stages rather than relying on a fixed image capture system to record gait motion. Furthermore, regarding neural networks, MLP has the advantage of having a low computational load relative to the other neural networks with reasonable accuracy for kinematic gait trajectory data prediction. However, with the availability of data, it performed much worse than CNN when there was a lack of input data, such as during the CGP. On the other hand, in CNN, the convolutional layers could extract the features of the sequential data even without providing any more information about the provided readings. This means that when using additional input such as the CGP, it did not have the same significant effect on the accuracy of the CNN (CC from 0.973 to 0.979) as it did on MLP (CC from 0.945 to 0.979), as seen in Table 6. However, as the accuracy of the CNN did not increase significantly, the load on the MC surged about five times, reaching 142 ms, while for the MLP, even with increasing accuracy, the load on the MC stayed at 42 ms, which is much faster than CNN.

**Figure 12:**
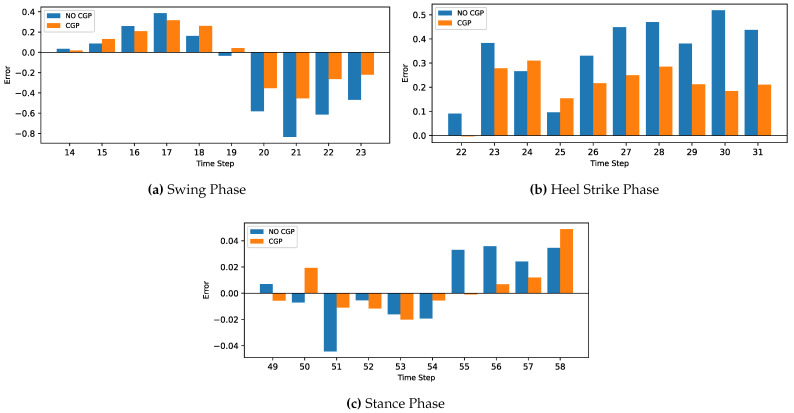
Comparison between two cases, with and without CGP for the MLP model.

**Figure 13:**
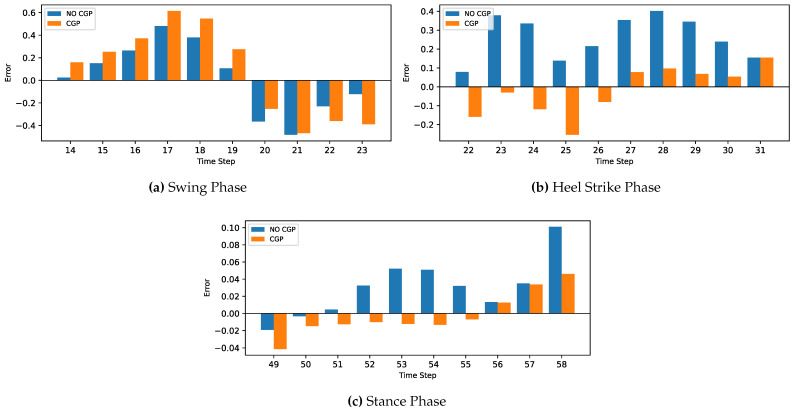
Comparison between two cases with and without CGP for the CNN model.

For the current study’s limitations, the training and testing were limited to five subjects only due to gyroscope data corruption, and that is not enough to generalize the trained model. In future work, a dataset will be used with more sensor readings such as force sensors and more subjects. In order to achieve better accuracy while maintaining the size of the network to counter what is lacking in the current approach, such as Figure 7, Figure 8, Figure 9, Figure 10, Figure 11, Figure 12 and Figure 13, the following points have to be solved:

- Phase shift during swing phase, which is more obvious in CNN for both with and without CGP.
- Initial jump in the prediction especially in CNN.

## 6. Conclusions

The developed MLP and CNN can overall predict the trajectory of the foot angular velocity using angular velocity and linear acceleration data from the shank only for multiple time frames ahead, with higher accuracy compared to LSTM, which is proven to be reliable regarding sequential data. Furthermore, when comparing MLP and CNN, it was found that CNN has the ability to be more reliable than MLP with the minimum amount of data, which can be helpful in the case of scarce training data. Moreover, when provided with sufficient data, the MLP and CNN were close in prediction accuracy. Yet, the MLP network had a lower computational inference time than CNN, which can be critical in an embedded application, as it can improve the controller’s delay and achieve a smooth transition between gait phases when used in an active prosthesis such as an active lower limb or exoskeleton. Additionally, the accuracy can be improved by using more sensors reading in addition to the IMU sensor. For example, a grid of force sensors will provide enough data to improve the neural network’s learning.

## Figures and Tables

**Table 1:** Left Limb Kinematic Parameters.

Limb	Sensor	Axis
Shank	Accelerometer	*X*-axis
Shank	Accelerometer	*Z*-axis
Shank	Gyroscope	*Y*-axis
Foot	Gyroscope	*Y*-axis

**Table 2:** DMLP model architecture.

Layers	Type	Number of Neurons
0	Input layer	1 × 20
1	Dense layer	100
2	Batch normalization layer	-
3	Dense layer	50
4	Dropout layer	0.01
5	Dense layer	30
6	Batch normalization layer	-
5	Dense layer	20
6	Output layer	10
-	Activation function	ReLU
-	Learning rate	0.001
-	Cost function	Mean Absolute Error

**Table 3:** DCNN model architecture.

Layers	Type	Number of Neurons	Convolutional Kernel Size
0	Input layer	1 × 20 × 1	-
1	Convolution layer	1 × 20 × 128	2 × 2
2	Max-pooling layer	1 × 8 × 128	2 × 2
3	Convolution layer	1 × 8 × 64	2 × 2
4	Max-pooling layer	1 × 4 × 64	2 × 2
5	Flatten layer	-	-
6	Dense layer	30	-
7	Dropout layer	0.01	-
8	Dense layer	20	-
9	Dropout layer	0.01	-
10	Dense layer	10	-
11	Dense layer	5	-
12	Output layer	10	-
-	Activation function	ReLU	-
-	Learning rate	0.001	-
-	Cost function	Mean Absolute Error	

**Table 4:** Models normalized statistical results comparison.

	Without CGP		With CGP	
Parameter	MLP	CNN	MLP	CNN
Bias (deg/s)	−0.849	−0.406	−0.095	−0.451
MAE (deg/s)	0.209	0.166	0.153	0.150
RMSE (deg/s)	0.298	0.245	0.226	0.217
R^2^	0.917	0.944	0.952	0.956
CC	0.945	0.973	0.979	0.979

**Table 5:** Microcontroller neural network testing.

	Without CGP		With CGP	
Parameter	MLP	CNN	MLP	CNN
Inference Time	2.237 ms	27.327 ms	2.427 ms	142.072 ms
Ram	13%	13%	13%	17%
Memory	11%	11%	11%	11%

**Table 6:** Results Comparison.

	Su Binbin	Zroug	Achieved Results (CGP)	
Parameter	LSTM	ED-LSTM	MLP	CNN
Bias (deg/s)	0.005	-	−0.095	−0.451
MAE (deg/s)	0.299	0.276 ± 0.14	0.153	0.150
RMSE (deg/s)	0.487	0.419 ± 0.25	0.226	0.217
R^2^	-	-	0.952	0.956
CC	0.91	0.89 ± 0.14	0.979	0.979

## Data Availability

The dataset used accessed on 15 April 2022: https://github.com/romanchereshnev/HuGaDB.

## Abbreviations

The following abbreviations are used in this manuscript:

ANN	Artificial Neural Network
biLSTM	Bi-directional Long Short-Term Memory
CC	Correlation Coefficient
CGP	Current Gait Phase
CNN	Convolutional Neural Network
DCNN	Deep Convolutional Neural Network
DMLP	Deep Multi-Layered Preceptron
GRF	Ground Reaction Force
IMU	Inertial Measurement Unit
LSTM	Long Short-Term Memory
MAE	Mean Absolute Error
MAF	Moving Average Filter
MCU	Microcontoller
MDPI	Multidisciplinary Digital Publishing Institute
MLP	Multi-Layered Preceptron
RMSE	Root Mean Squared Error
ST	Stance Phase
SW	Swing Phase
TOF	Time of Flight
TS	Time Step
